# Complete Genome Sequence Analysis of a Naturally Reassorted Infectious Bursal Disease Virus from India

**DOI:** 10.1128/genomeA.00709-16

**Published:** 2016-07-21

**Authors:** P. Raja, T. M. A. Senthilkumar, M. Parthiban, A. Thangavelu, A. Mangala Gowri, A. Palanisammi, K. Kumanan

**Affiliations:** a Department of Animal Biotechnology, Madras Veterinary College, Tamil Nadu Veterinary and Animal Sciences University, Chennai, India; b Department of Veterinary Microbiology, Madras Veterinary College, Tamil Nadu Veterinary and Animal Sciences University, Chennai, India; c Faculty of Basic Sciences, Madras Veterinary College, Tamil Nadu Veterinary and Animal Sciences University, Chennai, India

## Abstract

The novel infectious bursal disease virus (IBDV) isolate BGE14/ABT1/MVC/India is a very virulent IBDV that was isolated from broiler flocks in southern parts of India during 2014. Here, we report, for the first time in India, the complete genome sequence of BGE14/ABT1/MVC/India, a reassortment strain with segments A and B derived from a very virulent IBDV strain and an attenuated IBDV, respectively. The findings from this study provide additional insight into the genetic exchange between attenuated and very virulent strains of IBDV circulating in the field.

## GENOME ANNOUNCEMENT

Infectious bursal disease (IBD) is a highly contagious disease of young chickens caused by IBD virus (IBDV) belonging to the *Birnaviridae* family (1). Two serotypes have been recognized to naturally infect chickens. In the pathogenic serotype I, the large genomic segment A encodes for four viral proteins, the two capsid proteins VP2 (48 kDa) and VP3 (32 to 35 kDa), the viral protease VP4 (24 kDa), and a nonstructural protein VP5 (17 to 21 kDa). The smaller segment B encodes for the RNA-dependent RNA polymerase VP1 protein (94 kDa) (2). Coinfection with very virulent IBDV (vvIBDV) and attenuated IBDV strains has led to the exchange of double-stranded genomic RNA segments to generate new reassortment viruses (3–5).

The BGE14/ABT1/MVC/India strain was isolated from four-week-old commercial broiler flocks in India. This isolate caused immunosuppression, low weight gain, and bursal atrophy with high mortality. The presence of IBDV was diagnosed by agar gel immunodiffusion using specific hyperimmune serum and reverse transcriptase (RT)-PCR by amplifying the VP2 hypervariable region (6). The complete genome sequencing was carried out by RT-PCR using overlapping consensus primers and Sanger’s dideoxy sequencing in both directions by M/s Shrimpex Biotech, Chennai, India. Sequences were compiled and edited using the SeqMan program (Lasergene). Multiple sequence alignments were performed with MEGA5 (7), and a phylogenetic tree was constructed using PhyML.

The complete genome of segment A showed the highest nucleotide similarity (99.4%) with vvIBDV strain SH-99 (LM651365). In segment B, the highest similarity (99.9%) was found with an attenuated strain D78 (AF499930). The phylogenetic analysis indicated that segment A formed a cluster with vvIBDV strains, while segment B formed a cluster with attenuated and classical virulent reference strains. The results revealed that segment A of BGE14/ABT1/MVC/India was derived from a vvIBDV strain and that segment B was derived from an attenuated strain (8). There were three unique amino acid (aa) substitutions (L74I, P125S, and W133R) in VP5 compared to other vvIBDV isolates. The difference observed in the polycationic C-terminal region (aa residues 132 to 143) might be responsible for the variation in virulence and adaptability (9).

Analysis of the VP2 sequence of IBDV showed that there are four unique aa residues—222(A), 242(I), 256(I), and 299(S)—for the vvIBDV strains. Isoleucine was substituted by valine at residue 294(I-V). The serine-rich heptapeptide positions from aa 326 to aa 332 (SWSASGS) were found conserved, indicating that this isolate belonged to vvIBDV subtypes (10). The VP1 protein of the vvIBDV strains contains 15 characteristic amino acid residues (11). In contrast, the BGE14/ABT1/MVC/India strain carried the differences in all of the amino acid residues (V4I, K13T, I61V, T145N, D146E, N147G, E242D, A287T, M390L, D393E, K508R, S511R, P562S, P687S, and P695K), which made it identical to most of the classical, variant, and attenuated IBDV strains. These data are useful for analyses of epidemiology and evolutionary characteristics of IBDV in India.

### Nucleotide sequence accession numbers.

The full-length sequence of the BGE14/ABT1/MVC/India isolate has been deposited in GenBank with accession numbers KT884452 (segment A) and KT884453 (segment B).
